# Evaluation of Magnetic Micro- and Nanoparticle Toxicity to Ocular Tissues

**DOI:** 10.1371/journal.pone.0017452

**Published:** 2011-05-26

**Authors:** Hemalatha B. Raju, Ying Hu, Anil Vedula, Sander R. Dubovy, Jeffrey L. Goldberg

**Affiliations:** Bascom Palmer Eye Institute, University of Miami Miller School of Medicine, Miami, Florida, United States of America; Johns Hopkins School of Medicine, United States of America

## Abstract

**Purpose:**

Magnetic nanoparticles (MNPs) may be used for focal delivery of plasmids, drugs, cells, and other applications. Here we ask whether such particles are toxic to ocular structures.

**Methods:**

To evaluate the ocular toxicity of MNPs, we asked if either 50 nm or 4 µm magnetic particles affect intraocular pressure, corneal endothelial cell count, retinal morphology including both cell counts and glial activation, or photoreceptor function at different time points after injection. Sprague-Dawley rats (n = 44) were injected in the left eye with either 50 nm (3 µl, 1.65 mg) or 4 µm (3 µl, 1.69 mg) magnetic particles, and an equal volume of PBS into the right eye. Electroretinograms (ERG) were used to determine if MNPs induce functional changes to the photoreceptor layers. Enucleated eyes were sectioned for histology and immunofluorescence.

**Results:**

Compared to control-injected eyes, MNPs did not alter IOP measurements. ERG amplitudes for a-waves were in the 100–250 µV range and b-waves were in the 500–600 µV range, with no significant differences between injected and non-injected eyes. Histological sectioning and immunofluorescence staining showed little difference in MNP-injected animals compared to control eyes. In contrast, at 1 week, corneal endothelial cell numbers were significantly lower in the 4 µm magnetic particle-injected eyes compared to either 50 nm MNP- or PBS-injected eyes. Furthermore, iron deposition was detected after 4 µm magnetic particle but not 50 nm MNP injection.

**Conclusions:**

Intravitreal or anterior chamber injections of MNPs showed little to no signs of toxicity on retinal structure, photoreceptor function or aqueous drainage in the eye. Our results suggest that MNPs are safe for intraocular use.

## Introduction

Nanotechnology is an exciting new platform for translating advances in the basic sciences to therapeutics for eye disease. The nanoscale size of particles may confer advantageous properties for applications in drug delivery, gene therapy and cell and tissue engineering. Nanoparticles are well-suited to provide sustained drug delivery or gene therapy by virtue of highly controllable surface-area to volume ratios and improved tissue penetration [1].

Delivering drug, genetic, or cellular therapeutics efficaciously to internal ocular structures is a persistent challenge in ophthalmology. For example, drug delivery to the retina is limited by inadequate scleral tissue penetration, and recently increasing use of intravitreal injections leads to patient inconvenience and increased infection risk. Cell replacement therapies are limited by inadequate methods both to deliver cells to the targeted ocular tissue and then to retain the transplanted cells until integration. Hence, accurate and controllable delivery mechanisms are needed. Nanoparticle-based therapeutics may provide one such approach.

A number of materials have been studied for use in nanoparticle delivery systems, such as fibrin, gelatin, collagen, poly-lactic acid (PLA) and poly-lactide-co-glycolide (PLGA). We have been studying the use of magnetic nanoparticles (MNPs) which, in the presence of externally applied magnetic fields, can be used to control particle delivery and cellular localization and growth. MNP-bound cells, RNA and DNA can be separated in both research and clinical applications, including as a contrast agent in MRI [2], and as targeted delivery systems [3], [4], [5] and to apply mechanical forces to tissues or cells [6], [7].

It is unknown if MNPs cause toxicity to the eye, which could potentially limit their utility, nor whether use of particles on the nano scale confers any advantage or disadvantage compared to particles on the micron scale. Here we examine nanoparticle toxicity after injection into the eye either intravitreally or into the anterior chamber. By a variety of measures over the course of 5 months, nanoparticle-specific toxicity was undetectable, although there was a mild toxicity of microparticles, including corneal endothelial cell loss; iron deposition; and particle persistence out to 5 months.

## Results

To determine if either nano- or micro-magnetic particles induce ocular toxicity, we injected 50 nm and 4 µm magnetic particles either intravitreally (IVT) or into the anterior chamber (AC). Immediately after the injection, no immediate post-surgical ocular damage (e.g. intraocular bleeding) was observed, nor were there any behavioral abnormalities suggesting post-surgical stress in any of the groups. Intraocular pressure was measured one hour post-injection (see below) for all injected eyes. Injected animals were allowed to survive for 1 hour, 1 week, 1 month and 5 months, at which point they were euthanized and their eyes processed for histology (Table 1, and see below).

**Table 1:** Experimental groups.

Injection	Left Eye	Right Eye	Survival
IVT	50 nm	PBS	1 hour
IVT	4 µm	PBS	1 hour
IVT	50 nm	PBS	1 week
IVT	4 µm	PBS	1 week
IVT*	50 nm	PBS	1 month
IVT*	4 µm	PBS	1 month
IVT	50 nm	PBS	5 months
IVT	4 µm	PBS	5 months
AC	50 nm	PBS	1 hour
AC	4 µm	PBS	1 hour
AC	50 nm	PBS	1week
AC	4 µm	PBS	1week
AC*	50 nm	PBS	1 month
AC*	4 µm	PBS	1 month
AC	50 nm	PBS	5 months
AC	4 µm	PBS	5 months
None**			

3 animals were studied per group, but IOP and histology data for PBS-injected right eyes were pooled into groups irrespective of left eye treatment.

*1 month survival groups were only subjected to histology and iron staining, not IOP or immunofluorescence measurements.

**A set of uninjected animals was used exclusively for ERG measurements. IVT intravitreal; AC anterior chamber; PBS phosphate buffered saline.

### Intraocular pressure

**Figure 1:**
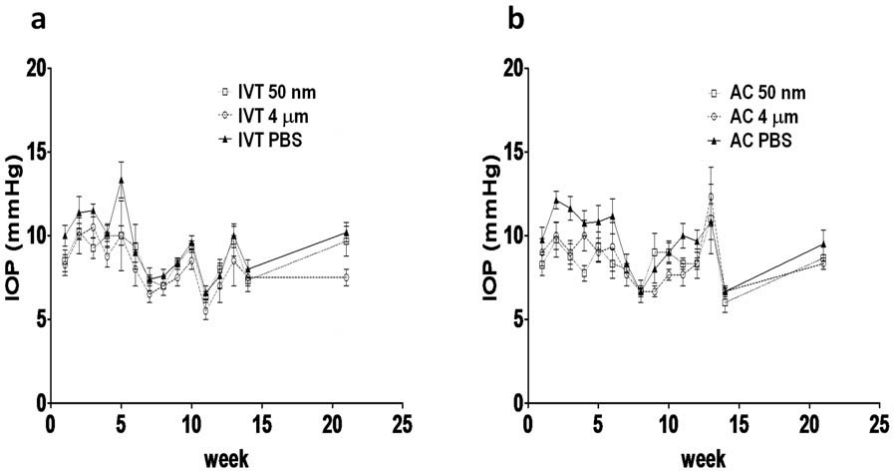
Intraocular pressure measurements. Intraocular pressure was measured using the rat tonolab for the (a) intravitreal (IVT) and (b) anterior chamber (AC) injections of 50 nm and 4 µm magnetic particles, and PBS. Measurements were made before injection, 1 hour after injection, 1 week after and every week thereafter until the animals were sacrificed. There were no significant differences between injected and control at any time point measured.

We first asked whether injection of magnetic particles led to an increase in intraocular pressure (IOP). From either IVT or AC injection, it is possible that magnetic particles could clog up the trabecular meshwork, leading to decreased aqueous outflow and increased IOP. We measured IOP using the rat tonolab immediately before and 1 hour after the injection, one day after, and every week until the animals were sacrificed. We found that the IOP for all three groups (50 nm, 4 µm and PBS) varied across the survival period, but at no time did any of the groups differ significantly from the others (Figure 1). There was a slight increase in IOP at week 5 for the PBS IVT injections (Figure 1a), but this never went above 15 mmHg, and was not statistically significant when compared to IOP in these same animals between weeks 5–10, or when compared to IOP in the magnetic particle-injected animals. The same was true with the AC PBS-injected animals in the first few weeks (Figure 1b). Slight trends seen in the data were thus more likely due to fluctuation in tonometer calibration or diurnal variability, as the statistics did not support any differences. We did not examine IOP immediately after injection, when human IVT injection is often found to increase IOP, although our slow injection rate may also differ from typical human IVT injections. Thus, neither nano- nor micro-particles induce significant changes in IOP compared to PBS control injections.

### Histochemistry and cell survival

**Figure 2:**
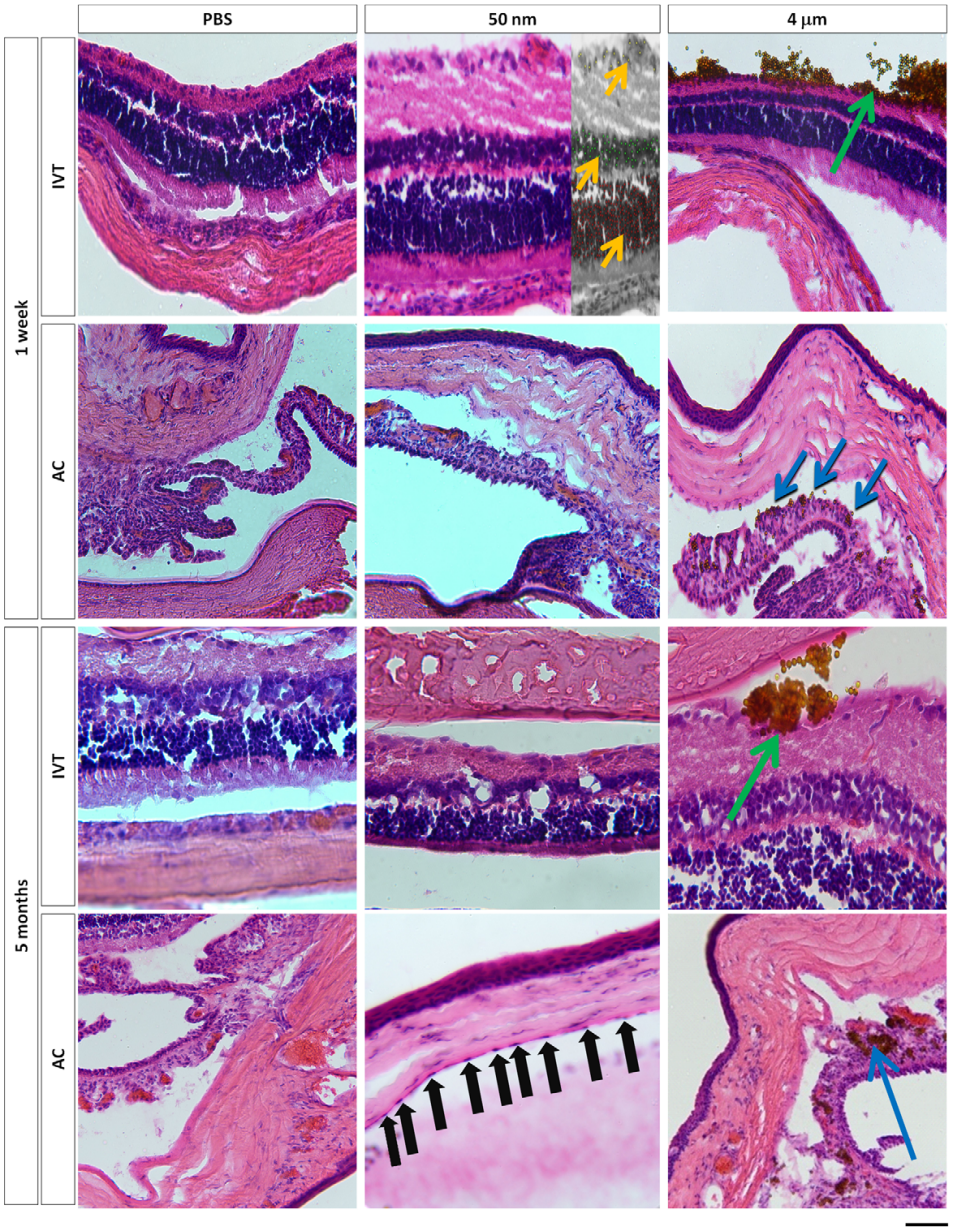
Hematoxylin- and eosin-stained sections. Hematoxylin- and eosin-stained representative sections for the PBS- (control), 50 nm- and 4 µm- injected animals at 1 week and 5 months, as marked. Accumulation of 4 µm particles were noted layered against the retina (IVT injection, green arrows) and along the iris and in the angle (AC injection, blue arrows) out to 5 months. Yellow arrows highlight example dotted cell nuclei in the GCL (yellow dots), INL (green dots) and ONL (red dots) at higher magnification used for cytotoxicity counting. Black arrows point to corneal endothelial cells along the endothelial cell layer, used for cytotoxicity counting. Scale bar, 50 µm in all pictures.

Next we asked if the IVT or AC injections of magnetic particles affected retinal or corneal endothelial cell numbers, respectively. Sections were processed for H&E histochemistry (Figure 2) and the number of cells in the ganglion cell layer (GCL), inner nuclear layer (INL) and outer nuclear layer (ONL) or in the corneal endothelial cell layer were counted. After AC injection, 4 µm particles were detected along the iris and in the angle (Figure 2 - blue arrows). After IVT injection, 4 µm particle clusters were detected layering the retina (Figure 2 - green arrows). In contrast to the 50 nm MNPs that were cleared from the vitreous in these experiments, 4 µm particles were still detectable at 5 months.

**Figure 3:**
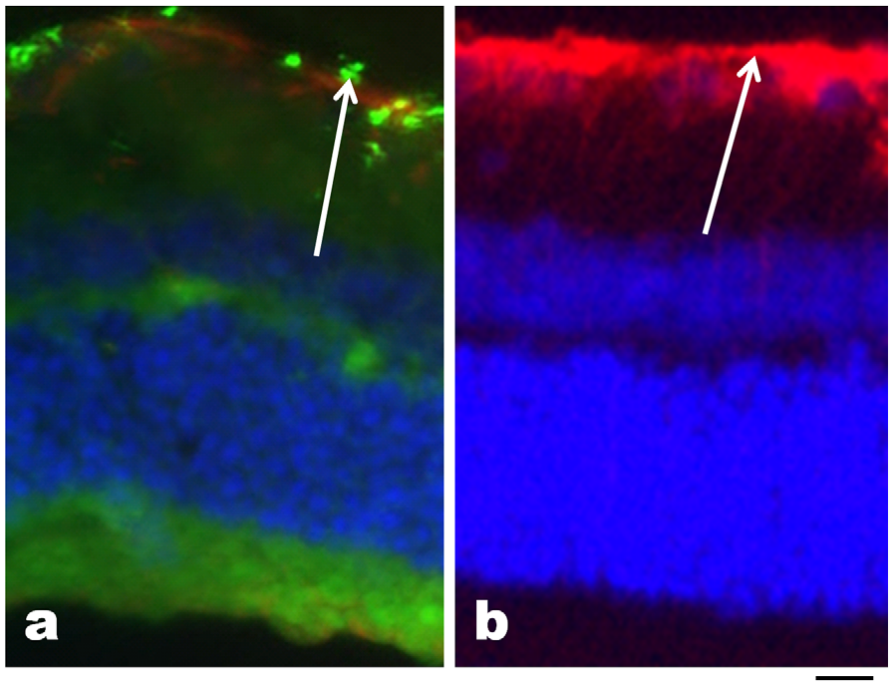
Localization of magnetic nanoparticles at 1 week. At 1 week after IVT injection, immunofluorescence was used to localize 50 nm particles (green in a) and 4 µm particles (red in b) as shown by white arrows. In both cases, retinas were counterstained with GFAP and DAPI (nuclei, blue). At 1 week, both sizes of magnetic particles were detectable in the ganglion cell layer (arrows). Scale bar, 50 µm.

**Figure 4:**
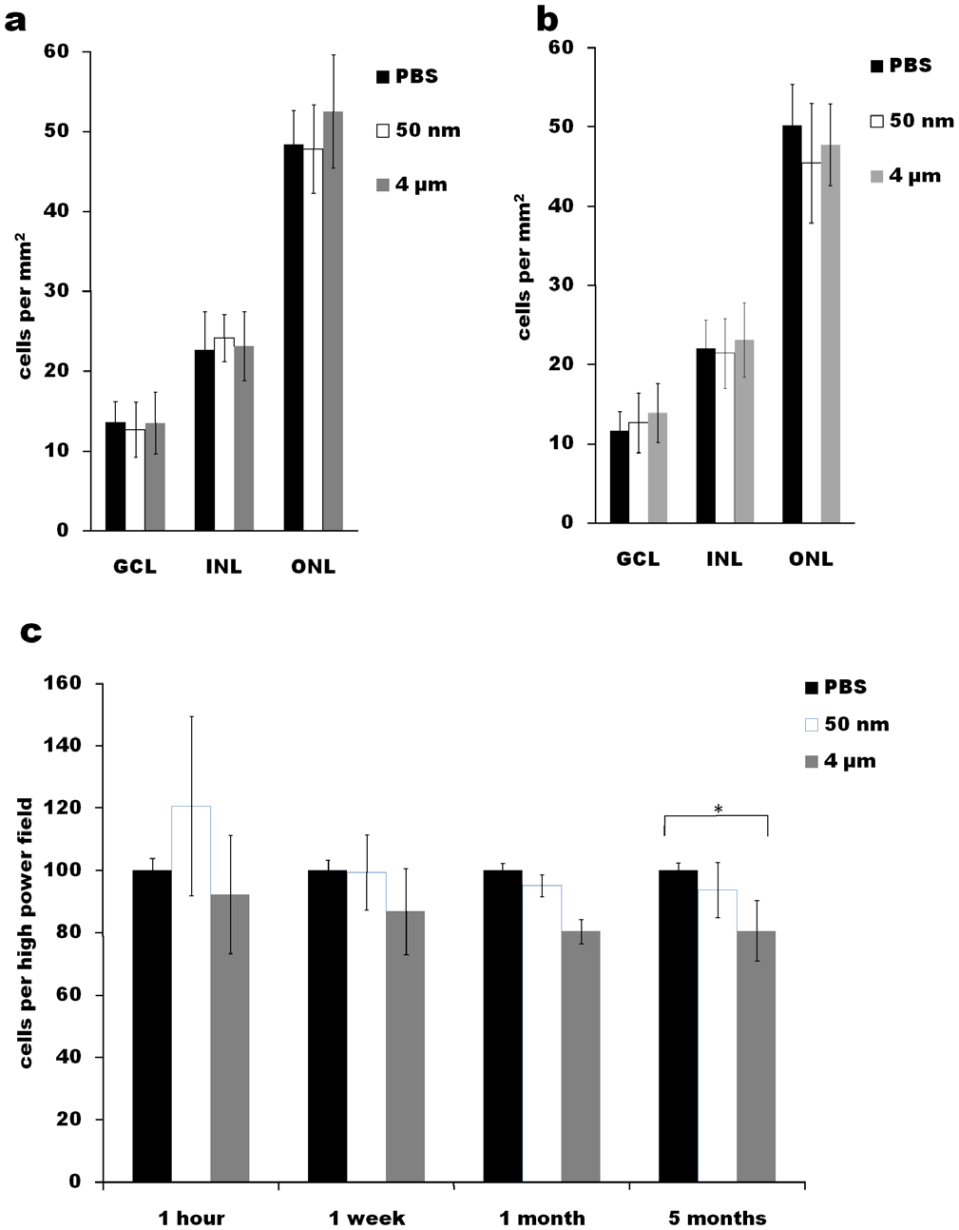
Cell count by histology after particle injections. Retinal layer cell numbers were quantified at (a) 1 week and (b) 5 months for ganglion cell layer (GCL), inner nuclear layer (INL) and outer nuclear layer (ONL) for the PBS control- and magnetic particle-injected eyes. (c) Corneal endothelial cell counts were normalized to PBS control-injected eyes for each time point shown. * p<0.05 by ANOVA and post-hoc t-test.

We could not detect 50 nm MNPs in histologic sections, but they were visible acutely after IVT injection in the GCL of the retina. At 1 week, 50 nm MNPs were detected without significant GFAP immunoreactivity (Figure 3a), whereas microparticles elicited significant GFAP upregulation (Figure 3b). No GFAP staining was detected in either group at 5 months (not shown). We found that there was no difference in the retinal nuclear cell density as measured along the GCL, INL and ONL at any time point from 1 week to 5 months (Figure 4a–b). Interestingly, we found that corneal endothelial cell numbers were no different between the control and 50 nm MNP AC-injected eyes, but there was a statistically significant decrease in corneal endothelial cell number in the eyes injected with 4 µm magnetic microparticles at 1 week and 5 months compared to PBS- and MNP-injected eyes (Figure 4b). Thus, 50 nm MNPs failed to detectably alter ocular histology, but magnetic microparticles were slightly toxic to the corneal endothelium.

### Glial activation in response to magnetic particles

**Figure 5:**
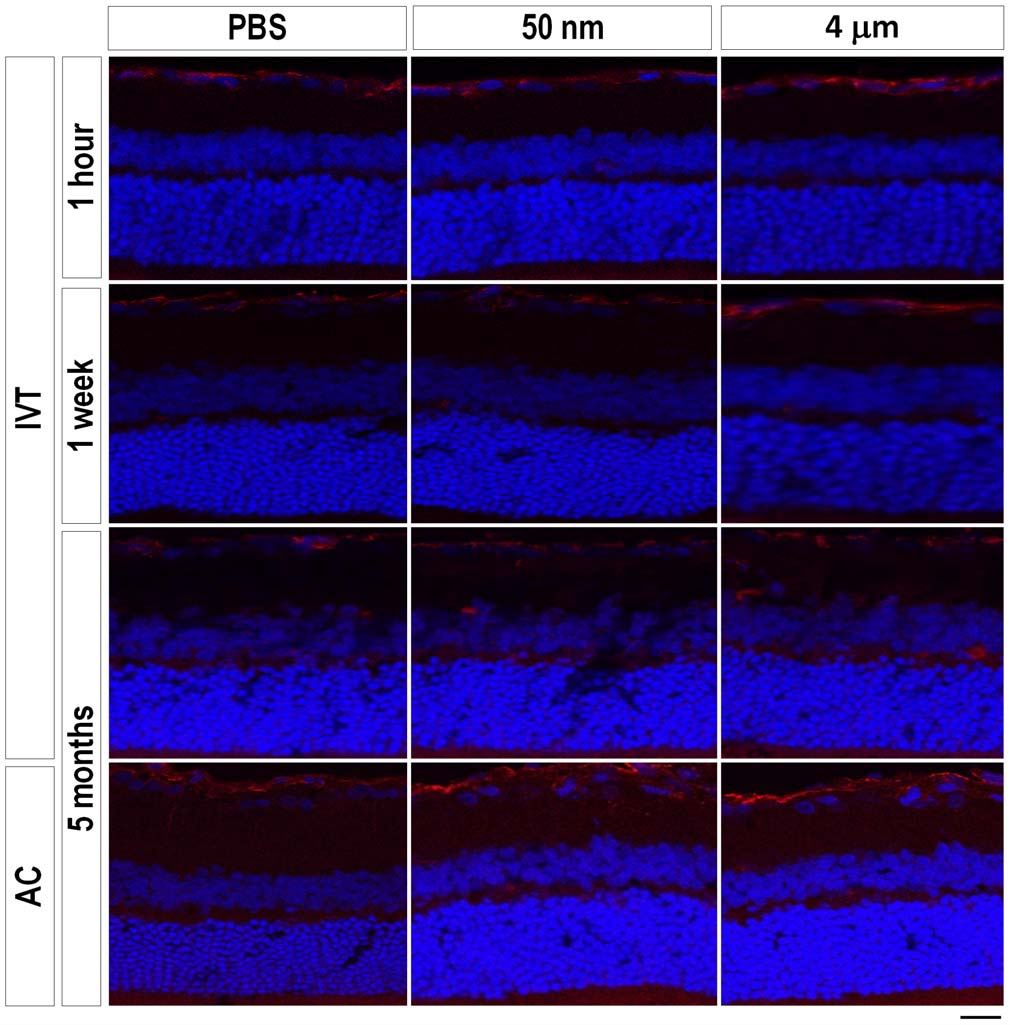
Measure of astrocyte activation using GFAP staining. Example GFAP immunofluorescence images for the PBS- (control), 50 nm- and 4 µm-intravitreally injected animals at 1 hour, 1 week and 5 months as marked. AC-injected animals are also shown for the 5 month time point. Astrocyte activation is seen along the ganglion cell layer (red) with nuclear counterstaining with DAPI (blue) highlighting the retinal layers. Astrocyte activation was similar among PBS- and magnetic particle-injected animals at all three time points for both types of injections. For IVT-injected animals, GFAP staining was seen primarily at the site of injection along the ganglion cell layer. Scale bar, 50 µm.

**Figure 6:**
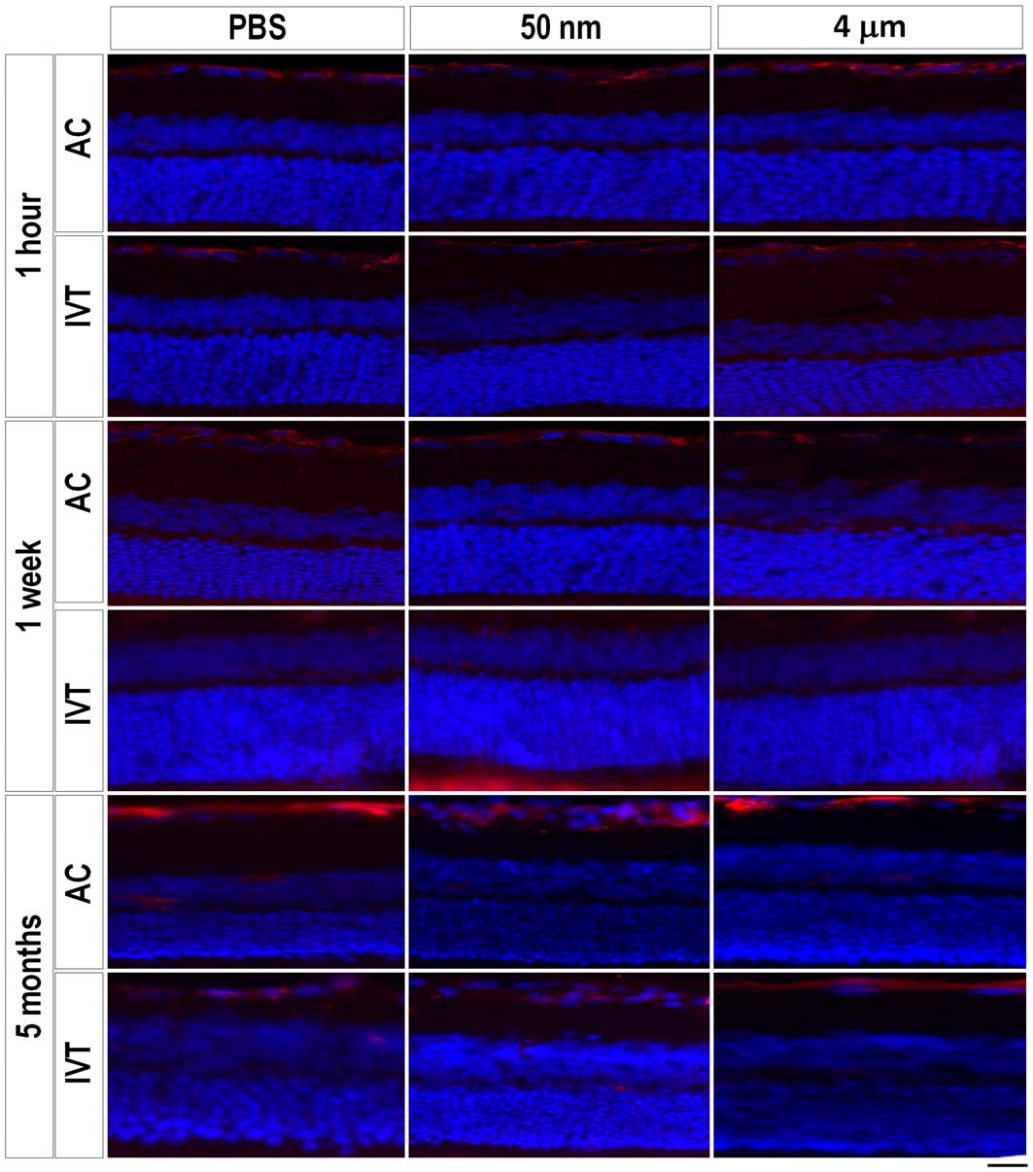
Microglial activation in response to magnetic particles. Example CD11b/c immunofluorescence images for the PBS- (control), 50 nm- and 4 µm-injected animals at 1 hour, 1 week and 5 months as marked. There was no difference in microglial activation among PBS- and magnetic particle-injected animals at any time point surveyed. Scale bar, 50 µm.

We next asked if magnetic particle injections induced astrocyte or microglial activation as measured by GFAP and CD11b/c immunofluorescence, respectively. We found GFAP immunoreactivity was similar in both PBS- and magnetic particle-injected animals at 1 hour, 1 week and 5 months, and that this minimal activation was mostly at the site of injection along the nerve fiber layer and GCL, without activation of Muller glia in the deeper retinal layers (Figure 5). A basal level of GFAP staining was seen in both AC- and IVT-injected animals at 5 months, suggesting that this level of GFAP immunoreactivity is background or at a minimum, not particle-specific. Similarly, there was no difference in CD11b/c staining among PBS- and particle-injected groups at 1 hour, 1 week and 5 months (Figure 6). Thus injection of magnetic nano- and microparticles do not appear to activate retinal glia.

### Iron deposition and electroretinograms

Next we asked if the injections of magnetic particles caused detectable iron deposition.

**Figure 7:**
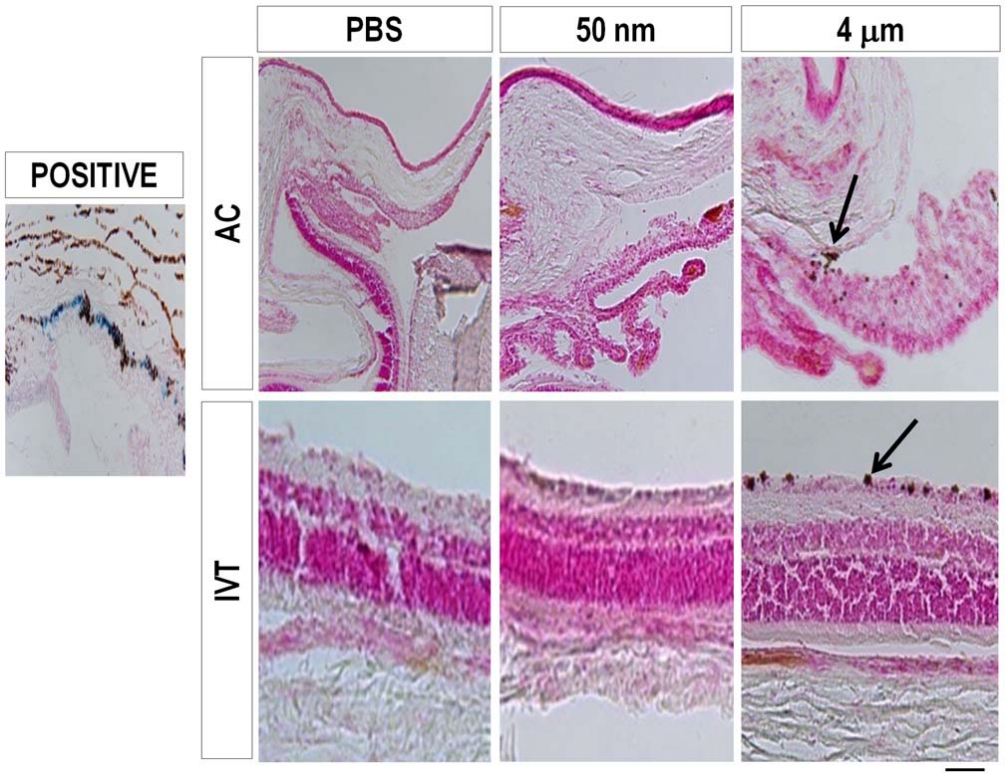
Iron deposits in the ocular tissues after particle injections. Representative images from Prussian blue histochemical staining for iron from control and magnetic particle-injected eyes at 1 week. Iron staining was observed only in the positive control, and not in any of the injected eyes, as marked. The 4 µm particles were visible in the iris and retinal tissues (arrows).

**Figure 8:**
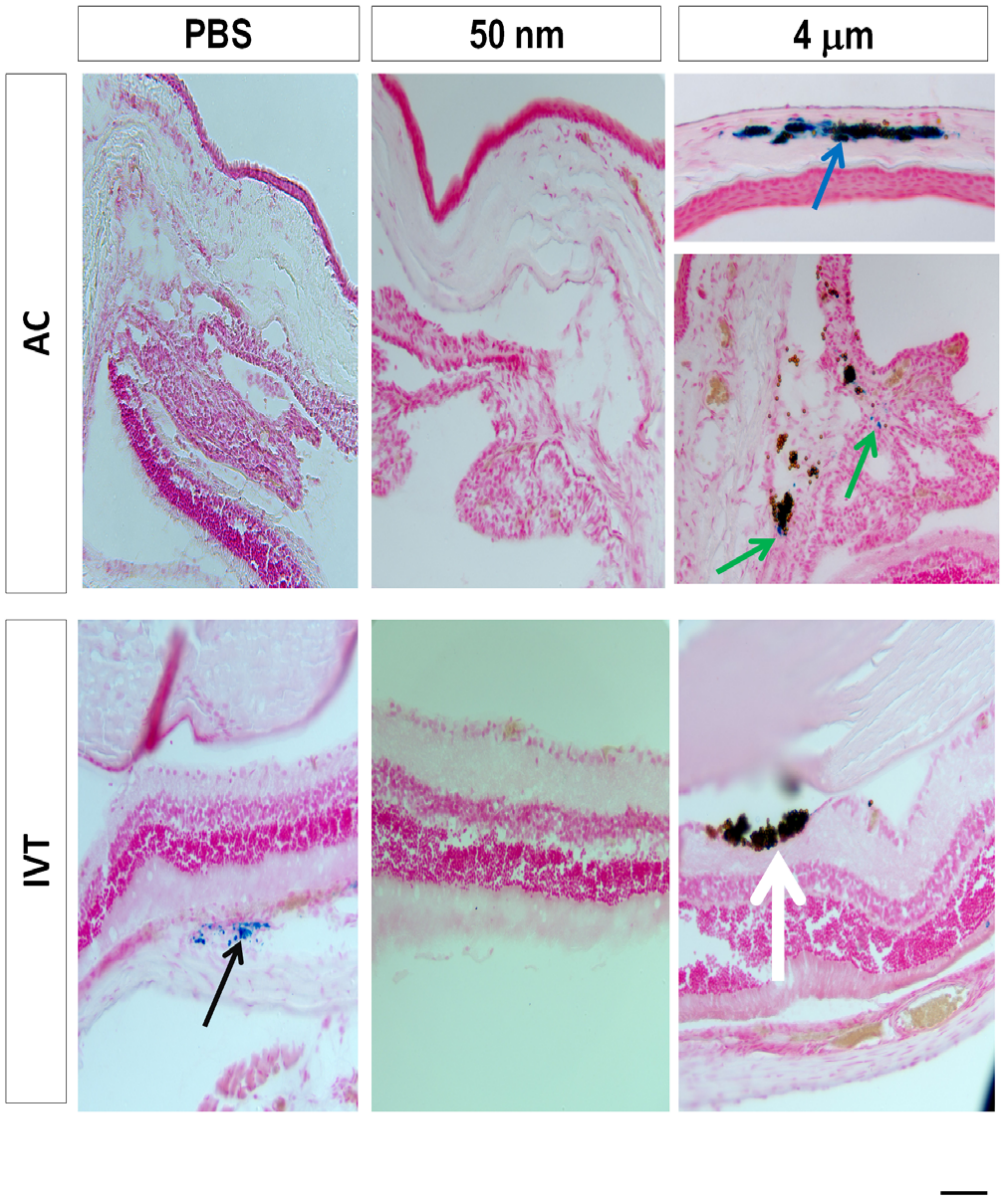
Iron deposits in the ocular tissues due to injected magnetic particles at 5 months. Prussian blue histochemical iron staining images for control and magnetic particle-injected eyes at 5 months. Black arrow shows an example of background choroidal staining in an IVT PBS- (control) injected eye, frequently observed as a background choroidal staining in many of the sections. Neither IVT nor AC 50 nm-injected eyes showed iron staining, but positive iron stains were noted around 4 µm particles after IVT injection (white arrow), in the cornea (blue arrow), and in the ciliary body (green arrows) seen after AC injection. Scale bar, 50 µm.

We stained histologic sections with Perl's Prussian Blue, and observed typical staining in the positive control tissues, as expected, but no staining was detected in any of the magnetic particle-injected eyes at 1 week (Figure 7). At 5 months after injection, iron staining was detected in the choroid for many of the animals, including in controls, which likely represents a normal feature of the aging choroid and thus was considered background (Figure 8). Other than this background choroidal staining, no iron staining was detected in any of the other ocular tissues after 50 nm MNP injections in the AC or vitreous. In contrast, in 4 µm particle injected eyes, iron staining was detected in the retina after IVT injection, and in the cornea and ciliary body after AC injection (Figure 8 and Table 2). Thus, by 5 months, iron deposition was only detected above background in the 4 µm particle-injected animals, but never in the 50 nm MNP-injected animals.

**Table 2:** Iron staining after particle injection – fraction of eyes staining with Perl's Prussian Blue.

		Choroidal	Other
	Control	0/3	0/3
50 nm	IVT	0/3	0/3
	Control (PBS)	0/3	0/3
	AC	1/3	0/3
	Control (PBS)	2/3	0/3
4 µm	IVT	3/3	2/3*
	Control (PBS)	1/3	0/3
	AC	0/2	2/2**
	Control (PBS)	1/2	0/2

*Staining seen around clumps of beads.

**Staining seen in cornea and ciliary body (Figure 8).

**Figure 9:**
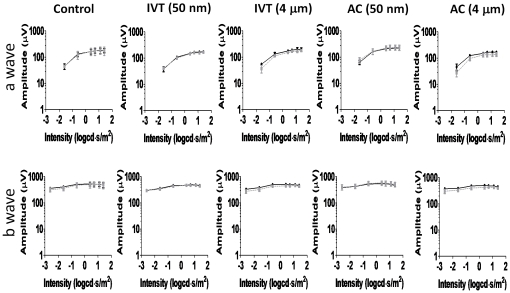
Measure of electroretinograms changes in response to magnetic particles. Electroretinograms taken at 9–14 weeks for 50 nm- and 4 µm-, AC- and IVT-injected animals, as well as control, uninjected animals, as marked. The solid line represents the average of the particle-injected left eyes for each group, and the dashed line represents the average of the PBS-injected right eyes for each group, except in the control animals (1^st^ column) in which neither eye was injected. There was no significant difference in the a- and b-waves for the control (uninjected) and nano- and microparticle injected animals with either IVT or AC injections.

Histochemical detection of ocular iron deposition may not be very sensitive, so we also examined the electroretinogram (ERG), which can be suppressed by molecular iron inside the eye [8], [9], [10]. We compared the PBS-injected right eyes to the magnetic particle-injected left eyes for all animals, and examined an additional set of animals in which neither eye was injected. We found similar amplitude ranges for all groups: a-waves in 100–250 µV range and b waves in 500–600 µV range (Figure 9). There were no significant difference in the a- and b-waves for the control (uninjected) and nano- and microparticle injected animals with either IVT or AC injections.

## Discussion

Here, we found that injecting magnetic nano- and microparticles into the vitreous or AC had essentially no particle-specific toxicity when measuring IOP, ERG, histology, or glial activation. We injected a single dose of magnetic particles 10-fold higher than a dose found to enhance RGC survival *in vitro* when coated with a TrkB agonist antibody (data not shown), which we previously demonstrated enhances RGC survival *in vitro* and *in vivo* [11], and consistent with other current uses of magnetic particles *in vitro* and *in vivo*. For example, one study in rat liver cells compared cellular toxic responses with respect to different sizes of nanoparticles with different core compositions and concluded that silver nanoparticles were highly toxic whereas molybdenum was moderately toxic and aluminium, iron oxide, manganese ozide and tungsten displayed little or no toxicity [12]. Similarly, IVT nanogold injections in rabbits showed few signs of retinal or optic nerve toxicity at 1 month, with only slight vacuolization in the inner plexiform and ganglion cell layers [13]. MNPs coated with polyethylenoxide copolymers are biocompatible as shown by a cell viability MTT assay and, up to a concentration of 5 mg/mL, do not disturb the growth of epithelial, endothelial or tumor cells [14]. The Food and Drug Administration (FDA) has approved the use of superparamagnetic iron oxide nanoparticles similar to those we used as contrast agents in magnetic resonance imaging [15], supporting the premise that nanoparticles prepared using iron oxide are not significantly harmful towards humans. Similarly, we found no toxicity of nanoparticles detected at the level of histology or glial activation. There was toxicity of microparticles, however, with a small but statistically significant decrease in corneal endothelial cell counts, as well as iron deposition in the tissues above control levels after AC injection (see below). Thus, magnetic nanoparticles, but not magnetic microparticles, were safe for intraocular injection, at least at the single injection dose tested. Note, magnetic fields were not applied to the MNP-injected eyes; it remains possible that MNPs may be more toxic if exposed to magnetic fields.

Using uncoated MNPs (not studied here) *in vivo* could be harmful, as it could lead to aggregation, increased oxidation, and or other instability in physiologic conditions. Adapting the surface of the particles can enhance biological compatibility and allow functionalization of the surfaces to deliver drugs or to bind receptors on target sites. For example, silica nanoparticle-associated toxicity can be reduced by synthesizing the particles with chitosan, a very well known biocompatible polymer [16]. The 50 nm MNPs used in our study were coated with dextran, a commonly used natural polymer that has been shown to be non-toxic and biocompatible [17], [18]. A limitation to this study was the difference in coatings between the two particle sizes, but both are well-described to be inert, biocompatible surfaces.

A major difference between nano- and microparticles in our study was the prolonged intraocular persistence of the microparticles. In the eye, the nanoparticles' clearance mechanism is unknown, but could involve uptake into the vascular system or passage through the hyaloid into the anterior chamber and egress out of the trabecular meshwork. After exiting the eye, the nanoparticles might be transported in lymphatic or systemic circulations and excreted in the liver and kidney [17], [18]. In contrast, the 4 µm particles persisted, presumably unable to exit through either of those pathways. We would expect longer time periods to lead to more iron leaching and iron-related toxicity from the 4 µm particles with longer time periods, given the persistence of those particles and their inability to leave the eye. We would not expect further toxicity from the 50 nm MNPs after 5 months, given that we were unable to detect them in the eye by this point. We argue that this supports a preference towards using nano- versus micron-scale particles in the eye for non-biodegradable and particularly iron-based particles.

Iron can damage photoreceptors and interfere with retinal electrophysiology [9] and ERG is a more sensitive and consistent modality with which to detect iron-related damage (siderosis) [8], [10] than Prussian blue staining. In a study in rabbits, ERG changes were detectable by 15 weeks [9], a similar time point to our study. We found that ERG waveforms did not show any difference in the amplitude between the magnetic particle and PBS injections. This could be attributable to the low total iron load delivered, or to the dextran and polystyrene coatings on the 50 nm and 4 µm particles, which could prevent molecular iron from leeching out. Coatings on MNPs may also confer toxicity, as for example decreasing polyethylenoxide (PEO; also called polyethylene glycol, PEG) tail lengths when coated on MNPs increases their toxicity in a series of cell lines including a human RPE cell line [14]. Thus coated nanoparticles are not toxic to photoreceptors at either the histologic or electrophysiologic levels.

Interestingly, we found that there was a consistent, albeit small, toxic effect of microparticles to the corneal endothelium, not seen with nanoparticles. This was detectable at 1 week and persistent out to 5 months, and correlated with detectable iron deposition found in the microparticle- but not nanoparticle-injected eyes. The corneal endothelium is particularly sensitive to insult in human eyes, for example after cataract surgery or in Fuch's endothelial dystrophy. We do not know why microparticles were more toxic than nanoparticles, but it may be due to physical trauma due to their larger size, or to the persistence of the microparticles inside the AC compared to the nanoparticles. After AC injection we were able to identify the 4 µm particles at 1 week and at 5 months (Figure 2 - blue arrows) lining the iris and angle, but even looking for the fluorescently-labeled nanoparticles, we could not find them at the same time points. It has been shown previously that particles ranging from 138 nm to 1.2 µm in diameter accumulated in the trabecular meshwork and demonstrated no correlation between the particle size and resistance to particle outflow [19], however these experiments did not examine 50 nm nanoparticles as we used here. We did not, however, find that magnetic microparticles led to an increase in IOP, suggesting that although they do not exit the eye efficiently, they did not clog the trabecular meshwork to a significant degree, possibly because of a difference in total number of particles injected. It has been shown that the injection of microbeads into the anterior chamber of rodents can be used to increase IOP and induce a rodent model of glaucoma [20]. Using 15 µm particles, they found that the increase in IOP was due to microbead obstruction of the trabecular meshwork, not seen here with 4 µm particles. It is possible the more subtle forms of cytotoxicity before cell loss measured here could be detected with other modalities like specular [21] or electron microscopy, and such studies may be warranted in the future.

In summary, polymer-coated MNPs and, to a slightly lesser degree, micron-scale particles, were largely non-toxic to ocular tissues whether injected IVT or into the AC. As such particles may prove to be excellent delivery vehicles for gene, drug or cellular delivery [1], understanding their interaction with ocular tissues will continue to be an important part of moving them towards potential therapeutic use.

## Methods

Experiments conformed to the ARVO Statement for the Use of Animals in Ophthalmic and Vision Research, and were approved by the Institutional Animal Care and Use Committee and the Institutional Biosafety Committee of the University of Miami (approval ID 08-094). For all survival experiments, animals were monitored including weekly weight measurements to confirm their health status. No animals were excluded due to weight loss or other health problems.

### Magnetic nano- and microparticles

Magnetic 50 nm nanoparticles (55–59% iron oxide w/v) are superparamagnetic, dextran-coated beads conjugated to goat anti-mouse IgG (Miltenyi Biotec Inc., Germany). Particle size is determined by passing the magnetic particles through a sieve/filter. Average radius was confirmed by dynamic light scattering using a DynaPro Titan Dynamic Light Scattering Instrument (Wyatt Technology, CA) with a mean particle radius of 33.6 nm and a polydisperisty of 0.372. To label these nanoparticles, 20 µl of nanoparticles were added to 20 µl of Alexa-488-conjugated donkey anti-goat IgG (Invitrogen) and incubated at room temperature for 10 min, and then centrifuged at 15.7 rcf for 10 min. The supernatant solution was discarded, the beads at the bottom of the tube were resuspended in 100 µl Dulbecco's phosphate buffered saline (DPBS), centrifuged at 15.7 rcf for 10 min, the supernatant discarded and nanoparticles resuspended in 20 µl DPBS, and then stored at 4°C for 1 day before injection.

Magnetic 4 µm particles (Dynabeads M-450, tosyl-activated, 37% iron oxide w/v) are superparamagnetic, polystyrene-coated particles (Invitrogen Dynal, Norway). Particle sizing is performed using a Coulter Counter Multisizer 3. The method is based upon pulses of current in an electrically conducting electrolyte. Briefly, the particles are dispersed in an electrolyte solution which is sucked into a tube through a small opening. When the particles pass through, the resistance across the opening is measured using a measuring cell coupled in a Wheatstone bridge. Each particle passing through the opening generates an electrical pulse, and the size of the pulse depends upon the particle size. The pulse sizes are measured and the particles are sorted accordingly. To label 4 µm particles, 20 µl of particles were added to 100 µl of buffer 2 (prepared according to the Dynal protocol, containing phosphate buffered saline (PBS), 0.1% bovine serum albumin (BSA) (w/v) and 2 mM EDTA, pH 7.4). The tube was placed in a magnet for 1 min and the supernatant was discarded. The particles were resuspended in buffer 1 (0.1 M sodium phosphate buffer) and 4 µg of Alexa-488-conjugated goat anti-rabbit IgG (Invitrogen), and then incubated at room temperature for one hour. The tube was placed in a magnet for 1 min and then the supernatant was discarded. The particles were washed twice with 1 ml buffer 2 and once with 1 ml buffer 2. The particles were resuspended in 20 µl buffer 2 and then stored at 4°C for 1 day before injection.

### Intraocular injections

36 Sprague Dawley rats (Harlan Laboratories) were anesthetized with ketamine (60 mgs/kg; 6 ml = 600 mg) and xylazine (8 mg/kg; 0.8 ml = 80 mg; dosage: 0.1 ml/100 grams of body wt.) and monitored throughout the procedure. The rats were injected intravitreally or in the anterior chamber with an equal volume of 3 µl of 4 µm (1.69 mg total) or 50 nm (1.65 mg total) magnetic particles in the left eye and an equal volume of 3 µl of PBS into the right eye as controls. This dose of particles was chosen because, based on an adult rodent vitreous volume of 52 µl [22], it was 10-fold higher than the dose found to enhance retinal ganglion cell (RGC) survival *in vitro* when coated with a TrkB agonist antibody (data not shown and [11]). This injection volume is also proportional to volumes currently injected into human eyes for clinical treatment today. Intravitreal injection was performed just posterior to the pars plana with a pulled glass micropipette attached to a 50 µl Hamilton syringe. Care was taken not to damage the lens. For anterior chamber injection, the cornea was punctured by inserting a 30-gauge needle directly above the pupil and parallel to the iris. Aqueous humor was allowed to flow out and was removed by sponge. 3 µl of MNPs was then injected with a pulled glass micropipette. At the end of the injection, an air bubble was introduced into the anterior chamber, to seal the corneal puncture and prevent leakage. There were none to very little adhesion of MNPs to the glass micropipette.

The animals were sacrificed 1 hour, 1 week, 1 month and 5 months after injection (Table 1). After the animals were sacrificed, the eyes were fixed with 4% paraformaldehyde and left overnight on sucrose. The eyes were frozen in liquid nitrogen and then sectioned at 16 µm thickness on a standard cryostat.

### Intraocular pressure measurements

Intraocular pressure (IOP) was measured using a TonoLab tonometer before the injection, one hour after the injection, one day after and every week after until the animals were sacrificed. Each eye was measured 3 times per session; reported results reflect the average of these 3 measurements.

### Immunofluorescence and histochemistry

Cryosections were blocked and permeabilized in 20% goat serum/0.2% triton X100 in antibody buffer (150 mM NaCl, 50 mM Tris base, 1% BSA, 100 mM L-Lysine, 0.04% Na azide, pH 7.4), for 30 min at room temperature. Primary antibodies against glial fibrillary acidic protein (GFAP) or CD11b/c (Abcam) for astrocytes and microglia/macrophages, respectively, were diluted in antibody buffer at 1∶500 and 1∶200 respectively. Sections were incubated in primary antibody solution overnight at 4°C, rinsed in PBS three times, incubated in Alexa-594-conjugated goat anti-mouse (Invitrogen) secondary antibody diluted in antibody buffer at 1∶400 overnight at 4°C ,rinsed in PBS three times, mounted in mounting medium with DAPI (Vector Laboratories Inc., CA) and imaged with an Axiophot microscope (Zeiss).

Alternating cryosections were stained with hematoxylin and eosin (H&E) using typical protocols to examine cellular morphology. Using the H&E-stained sections, cell density was measured in three retinal nuclear layers – ganglion cell layer, inner nuclear layer and outer nuclear layer – and in the corneal endothelial cell layer at all three time points (1 hour, 1 week and 5 months) and reported as cells per mm^2^ for retinal cells, and cells per linear mm for corneal endothelial cells. About 8 sections per eye were examined, with 3 eyes per group (Table 1). Cell counts were done by eye under microscopy; an example retinal section was marked in Figure 2(yellow arrow) using ImageJ (NIH) for demonstration purposes. About 10–12 sections per treatment were evaluated and averaged. Averages, standard deviations, and statistical analysis using ANOVA with post-hoc t-tests were calculated in Excel (Microsoft).

Alternating cryosections were stained with Perl's Prussian Blue using standard protocols to look for iron deposition in the ocular tissues at all 3 time points. These were examined qualitatively.

### Electroretinogram (ERG) recording

Animals at 9–14 weeks post-injection were dark adapted for 24 h and then anesthetized under dim light with ketamine and xylazine. Pupils were dilated with 0.1% atropine. During measurements, the animals were placed on a heated pad (37°C) to maintain body temperature. Custom contact lenses made with platinum wire and placed on the corneas of both eyes served as active electrodes, a subcutaneous needle electrode between the eyes was used as a reference electrode, and a subcutaneous needle electrode in the tail served as the ground electrode. All of the operations including anesthetizing animals, animal positioning and electrode placement were performed under dim red light, to preserve dark adaptation. ERG measurements were made using the UTAS Visual Diagnostic System (LKC Technologies). The Single Flash test type for the Sunburst Flash was used, with an amplifier gain of ±1250 v and bandpass filtered between 0.3 and 500 Hz. The time of measurement was 200 ms. Standard waveforms were recorded at seven different light intensities −30 dB, −20 dB, −10 dB, 0 dB, 2 dB, 5 dB, 10 dB (−2.6 to 1.3 log cd-s/m^2^) where 1 dB = 10log (cd-s/m^2^/2.5). The built-in smoothing algorithm was used for removing excess high frequency noise from a waveform. Using the EMWIN 8.1.1 software, amplitudes of a-wave were measured from the baseline to the bottom of the a-wave trough and b-wave amplitudes were measured from the bottom of the a-wave trough to the top of the b-wave. In the absence of a-wave, b-wave amplitude was calculated from the baseline to the peak of the b-wave. All animals were measured at 9–14 weeks. ERG results were averaged among each treatment group; means, standard errors and statistical significance (ANOVA and t-test) were calculated in Excel (Microsoft).
